# Location, location, location. *Salmonella* senses ethanolamine to gauge distinct host environments and coordinate gene expression 

**DOI:** 10.15698/mic2016.02.479

**Published:** 2016-01-18

**Authors:** Christopher J. Anderson, Melissa M. Kendall

**Affiliations:** 1 Department of Microbiology, Immunology, and Cancer Biology, University of Virginia School of Medicine, Charlottesville, Virginia, U.S.A.

**Keywords:** Salmonella, ethanolamine, macrophages, GI tract, metabolism

## Abstract

Chemical and nutrient signaling mediate all cellular processes, ensuring survival in response to changing environmental conditions. Ethanolamine is a component of phosphatidylethanolamine, a major phospholipid of mammalian and bacterial cell membranes. Ethanolamine is abundant in the gastrointestinal (GI) tract from dietary sources as well as from the normal turnover of intestinal epithelial and bacterial cells in the gut. Additionally, mammalian cells maintain intracellular ethanolamine concentrations through low and high-affinity uptake systems and the internal recycling of phosphatidylethanolamine; therefore, ethanolamine is ubiquitous throughout the mammalian host. Although ethanolamine has profound signaling activity within mammalian cells by modulating inflammatory responses and intestinal physiology, ethanolamine is best appreciated as a nutrient for bacteria that supports growth. In our recent work (Anderson*, et al.* PLoS Pathog (2015), 11: e1005278), we demonstrated that *Salmonella enterica* serovar Typhimurium (*Salmonella*) exploits ethanolamine signaling to adapt to distinct host environments to precisely coordinate expression of genes encoding metabolism and virulence, which ultimately enhances disease progression.

Gram-negative and Gram-positive bacteria encode the ethanolamine utilization (*eut*) operon. This operon has been most widely studied in the *Enterobacteriaceae*, and several research groups established that this operon contains 17 genes encoding enzymes and a microcompartment necessary for the breakdown of ethanolamine (Figure 1A). The *eut* operon also encodes the transcriptional regulator EutR. Genetic studies by Roof and Roth showed that EutR is constitutively expressed at low levels from the P2 promoter, and in the presence of ethanolamine and the cofactor vitamin B_12_, EutR induces expression of the entire *eut *operon. Previously, we used biochemical approaches and demonstrated that EutR directly senses ethanolamine and binds to the *eutS* (P1) promoter to activate transcription (Figure 1A). Additionally, we reported that enterohemorrhagic *Escherichia coli* exploits ethanolamine signaling through EutR to activate expression of virulence genes *in vitro, *which established the importance of ethanolamine as a bacterial signaling molecule. However, the question remained whether ethanolamine signaling influences bacterial virulence *in vivo*. 

**Figure 1:**
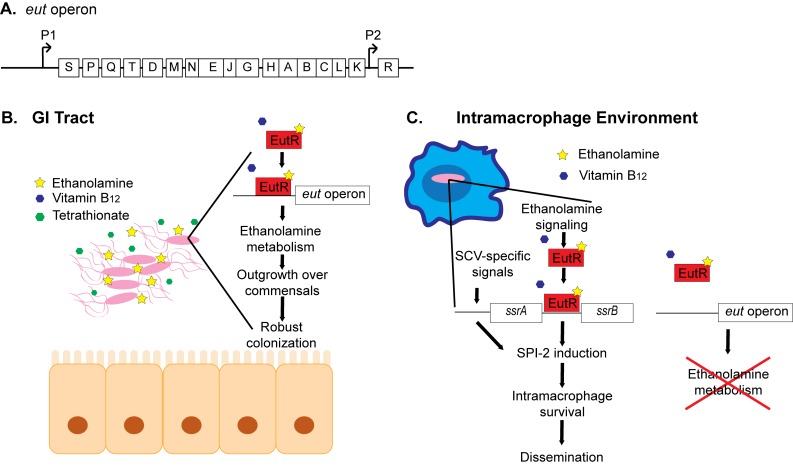
**(A)** Schematic of the *eut *operon. **(B)** In the GI tract, *Salmonella *- induced inflammation releases the electron acceptor tetrathionate. Here, EutR promotes expression of the *eut* operon, which enables *Salmonella* to outgrow the resident microbiota and establish infection. **(C)** Within macrophages, *Salmonella* relies on ethanolamine signaling through EutR, in conjunction with SCV-containing signals, to induce robust expression of SPI-2. This results in increased intracellular survival and subsequent dissemination. In macrophages, ethanolamine metabolism does not provide a growth advantage, and expression of the *eut* operon is not induced.

To address this, we investigated ethanolamine signaling in *Salmonella* pathogenesis. *Salmonella* is a food-borne pathogen that causes acute gastroenteritis. Although *Salmonella* infections are typically limited to the GI tract, a subset of infected individuals will develop systemic infections that can be fatal. *Salmonella* infection presents as intestinal outgrowth, penetration of the epithelial barrier, and subsequent uptake by macrophages, where *Salmonella *is ultimately trafficked to secondary lymphoid organs such as the spleen. To address whether ethanolamine signaling impacted *Salmonella* disease progression during infection, we performed competition experiments and examined recovery of wild type (WT) *Salmonella*, the Δ*eutR* strain that cannot respond to ethanolamine or the Δ*eutB* strain that cannot metabolize ethanolamine, from the intestinal contents, the colon, and the spleen. After orogastric infection of streptomycin-treated mice, the ∆*eutR* and ∆*eutB* strains were recovered in significantly lower numbers compared to WT from intestinal contents, indicating that ethanolamine metabolism is critical for *Salmonella* colonization of the GI tract. These results agreed with previous findings by the Bäumler lab that reported that *Salmonella* respires ethanolamine in conjunction with the inflammation-derived electron acceptor tetrathionate to outgrow the resident microbiota and establish infection. Significantly, examination of splenic tissue revealed that the ∆*eutB* strain was only slightly attenuated in dissemination to the spleen compared to WT, whereas the ∆*eutR* strain displayed a more robust disadvantage in dissemination to the spleen. To distinguish between ethanolamine metabolism-dependent and -independent roles for EutR* in vivo*, we directly competed the ∆*eutR* and the ∆*eutB *strains. No differences in the numbers of recovered bacteria were measured after 2 days post infection (dpi) in the intestinal contents, indicating that at this early stage and site of infection EutR functions primarily to direct ethanolamine metabolism. However, at 4 dpi the ∆*eutR* strain was significantly outcompeted by the ∆*eutB* strain in the intestinal contents. This time point is consistent with progression to systemic infection, and in agreement, the ∆*eutR* strain was recovered in significantly lower numbers compared to the ∆*eutB* strain from the spleen. These data indicated that the role of EutR during infection is dynamic and extends beyond promoting metabolism.

Intramacrophage survival and replication is a major means by which *Salmonella* disseminates, and we focused on the role of ethanolamine signaling on this aspect of *Salmonella* pathogenesis. To determine how EutR functions to promote dissemination, we assessed *Salmonella* survival within RAW and primary macrophages. Following macrophage infection, the Δ*eutR* strain was recovered at significantly lower numbers compared to WT or the Δ*eutB* strains, indicating that ethanolamine signaling through EutR enhances *Salmonella* survival within macrophages, in a manner independent from promoting metabolism.

Based on these findings, we hypothesized that EutR regulates expression of the *Salmonella* pathogenicity island (SPI)-2. SPI-2 contains four operons that encode a type three secretion system and effectors. Additionally, SPI-2 encodes a two component system (TCS), SsrAB, in which SsrA is the sensor kinase and SsrB is the response regulator that directly activates SPI-2 expression. Extensive research has established that SPI-2 expression is restricted to the intracellular environment and is essential for intramacrophage survival. Expression of *ssrB* and downstream SPI-2 operons was significantly decreased in the Δ*eutR* strain compared to WT *Salmonella* during macrophage infection, indicating that ethanolamine signaling plays an important role in *Salmonella* virulence regulation. Interestingly, although *ssrB* expression was decreased in the Δ*eutR* strain, EutR did not modulate *ssrA* expression. The *ssrAB* operon contains two promoters, one immediately upstream of *ssrA* and a second promoter immediately upstream of *ssrB*, which allow for *ssrB* expression independent of SsrA. *In vitro* assays using purified EutR in conjunction with *in vivo* assays revealed that EutR directly binds the *ssrB* promoter to activate SPI-2 expression. These findings indicate a more extensive role for EutR in *Salmonella* pathogenesis than previously appreciated, as a direct regulator of virulence traits.

Control of SPI-2 expression is complex and also includes additional regulatory systems, including the EnvZ-OmpR and PhoP-PhoQ TCSs. These TCSs respond to stimuli in the *Salmonella *- containing vacuole (SCV) (a modified phagosome within which *Salmonella* replicates in macrophages) to activate SPI-2 expression. Our findings suggest a model in which EutR-dependent activation of *ssrB* enables *Salmonella* to incorporate ethanolamine, an abundant and integral component of host cell membranes, with SCV-specific signals to efficiently and temporally regulate SPI-2 expression. Further investigation is required to understand the interplay between EutR and the EnvZ-OmpR/PhoP-PhoQ systems in promoting SPI-2 expression.

To further elucidate the dynamics of EutR-associated signaling, we assessed expression of the *eut* operon in WT *Salmonella* during macrophage infection. Expression of *eutR *was highly induced within macrophages compared to *Salmonella* grown in the absence of macrophages, confirming that the intramacrophage environment is sufficient to activate EutR-associated signaling. Notably, *eutR* expression was most highly induced at 3 hpi, and expression levels decreased as infection progressed. These data suggest that ethanolamine signaling is important for the initial adaptation of *Salmonella* to the intramacrophage environment. Surprisingly, despite robust *eutR* expression, we did not measure an induction of the *eut* operon (as determined by measuring *eutS* expression) within macrophages.

To confirm these findings within the complexities of the *in vivo* environment, we performed competition infections using an intraperitoneal infection model, which specifically examines SPI-2 mediated systemic disease. We assessed recovery of the Δ*eutR* and Δ*eutB* strains from the peritoneal cavity as well as within peritoneal phagocytes. The Δ*eutR* and Δ*eutB* strains were equally fit in the peritoneal cavity, which contained primarily extracellular *Salmonella*; however, the Δ*eutR* strain was significantly outcompeted by the Δ*eutB* strain within the resident phagocyte population. Subsequently, we performed single infections with WT and the ∆*eutR* strains and measured gene expression from *Salmonella* recovered from spleens. In agreement with the macrophage data, expression of *ssrB* was significantly decreased in the ∆*eutR *strain compared to WT. Moreover, although *eutR* expression was significantly induced in WT *Salmonella*, *eutS *expression was below detection limits. Altogether, these findings highlight that EutR directs gene expression specifically according to a particular environment.

Pathogens rely on precise and coordinated gene expression to successfully grow within a host and cause disease. Our recent work highlights a sophisticated mechanism in which *Salmonella* exploits ethanolamine to spatiotemporally control expression of genes encoding metabolism and virulence. During the initial stages of *Salmonella* infection, EutR activates expression of the *eut* operon. This promotes robust growth of *Salmonella* and establishment of infection (Figure 1B). In the intracellular environment where ethanolamine metabolism in not energetically favorable, we hypothesize that EutR preferentially targets genes required for macrophage adaptation (Figure 1C). How EutR differentially promotes gene expression according to location within a host is an ongoing area of research in our lab. Genes encoding ethanolamine metabolism are widely distributed among bacteria. Therefore, ethanolamine signaling may be a conserved strategy used by diverse pathogens to recognize and adapt to distinct host environments.

